# Secondary Hardening of a High-N Ni-Free Stainless Steel

**DOI:** 10.3390/ma15217505

**Published:** 2022-10-26

**Authors:** Nathalie Siredey-Schwaller, Pierre Charbonnier, Yudong Zhang, Julien Guyon, Olivier Perroud, Pascal Laheurte

**Affiliations:** Laboratoire d’Etude des Microstructures et de Mécanique des Matériaux (LEM3), CNRS UMR 7239, Université de Lorraine, Arts & Métiers ParisTech, F-57000 Metz, France

**Keywords:** high N stainless steel, precipitation, microstructure, phase transitions, SEM, TEM

## Abstract

High-N Ni-free stainless steels are used for their excellent mechanical properties combined with their high corrosion resistance, especially for biomedical applications. Even though it is well-known that secondary hardening during annealing after cold working has been observed in many materials, this phenomenon was not reported for these materials, one of the best known being Biodur108©, although numerous efforts have been made to increase its hardness. In this work, thermomechanical treatments at low temperature of cold-deformed Biodur108© were conducted to increase the hardness. Hardness as high as 830 Hv was obtained. For this material, the annealing of a deformed sample at intermediate temperature leads to a secondary hardening phenomenon. The mechanisms responsible for this secondary hardening were analyzed. It was found that for deformed samples, annealing at 575 °C leads to the formation of small Cr_2_N precipitates along grain boundaries and sub-grain boundaries, and simultaneously with a new body-centered cubic (BCC) phase that possesses a super structure. The newly formed phases have sub-micrometric grain sizes.

## 1. Introduction

High-nitrogen, nickel-free austenitic stainless steels are known to exhibit an excellent combination of high corrosion resistance and high mechanical properties [1]. Due to the absence of nickel allowing for biocompatibility and low cost, such alloys are excellent materials for medical applications. For specific purposes, higher hardness and strength levels are however sought. This may be obtained by increasing nitrogen concentration level [2,3,4]. This may also be obtained by cold working as studied by Talha et al. [5] in Fe–18Cr–12Mn–0.5N and by Balachandran et al. [4] in Fe–19Cr–18Mn–0.8N–0.2C. When austenite is metastable at room temperature, cold working may induce martensite, as reviewed by Murata et al. [6]. In Fe–18Cr–19Mn–0.39N, one way to increase yield strength is to hot-roll the sample (at a temperature of 900 °C) leading to the creation of some austenite with dislocations and mechanical twins, prior to tensile testing [7]. A TRIP effect then occurred during tensile testing, with the formation of the α’ martensite and mechanical twinning. As proposed by Behjati et al. [8] in Fe–18Cr–12Mn–0.44N–0.05C or by Rasouli et al. [9], another way to achieve good mechanical properties (high strength/high elongation) is via grain refining, consisting in cold-rolling the samples, inducing α’ martensite which is transformed by heating up to about 700 °C to 850 °C in order to obtain 1 μm or smaller austenitic grains. By cold rolling the alloy with the N highest concentration (0.44%), Behjati et al. [8] obtain a hardness that can reach about 750 Hv.

Nevertheless, as nitrogen is a strong austenite stabilizer, this cold working does not induce martensite in high N-level steels. This is interesting because, due to this austenitic phase, high-nitrogen stainless steels are not sensitive to ambient magnetic fields, even when deformed. These steels are then suitable to manufacture components of mechanical watch movement parts. In that case, it is expected that this material be easily machined or deformed while reaching strong yield strength thanks to some post-machined thermal treatment. A possible alloy is Biodur 108© alloy [10], initially designed for biomedical application. It is the one of the biomedical alloys having the highest yield strength. In this alloy, this strength is usually increased by cold working, as presented by Patnaik et al. [11]. In the technical data sheet [10], it is specified that this alloy is not hardenable by thermal treatment, but only by cold working.

### 1.1. Deformation Mechanisms

The deformation of austenite and hence the mechanical resistance are associated with various microstructural mechanisms according to the alloy composition and the value of the stacking fault energy (SFE). The latter strongly depends on the composition, in a complex way. This was confirmed by Gavriljuk et al. [12] who indicated that SFE does not vary monotonically with the N concentration. However, for Fe–15Cr–17Mn–XN, with X varying from 0.23 to 0.8, SFE increases with the N concentration, up to values as high as 40 mJ/m^2^, as indicated by Lee et al. [13]. A modeling of SFE was proposed by Dai Qi-Xun et al. [14] and Mosecker et al. [15]. For SFE values standing between 18 mJ/m^2^ and 38 mJ/m^2^, the creation of twins during deformation is expected, while for higher SFE values, deformation induces dislocation glides. However, special features were found to be attributed to specific N effects leading to prove that, for the Ni-free high N alloys, SFE is not the only parameter that influences deformation mechanisms. This was also deduced from the work on Fe–21Cr–23Mn–0.9N by Saller et al. [16]. Recently, Molnar et al. [17] reviewed the effects of inserting N atoms in austenite. In particular, they noticed that nitrogen suppresses the cross-slip of dislocations by introducing a lattice friction. This promotes the creation of planar dislocation arrays. For example, in the Fe–19Cr–19Mn–0.7N studied by Singh et al. [18], austenite deformation leads to the creation of fine slip lines. For Saller et al. [16], this phenomenon is assumed to be the main reason of the increase in the work hardening rate in these alloys. Another phenomenon occurs that may explain the formation of strictly planar dislocation slips: it is the nitrogen-assisted short-range ordering. When Cr content is high, some Cr-N short-range ordering (SRO) clusters can form, as reviewed by Mosecker et al. [19], influencing mechanical behavior. Furthermore, Terazawa et al. [20] showed that increase in the Mn concentration allows for the creation of dislocations cells due to the fact that the cross-slip of screw dislocations occurs more easily in these conditions. They came to this conclusion by comparing the Fe–25Cr–1.1N alloy and the Fe–21Cr–23Mn–0.9N one.

### 1.2. Effect of Temperature on Mechanical Strength

It is well-known that an increase in tensile test temperature up to 500 °C of high N austenitic alloys such as Fe–19Cr–19Mn–0.8/0.9N studied by Li et al. [2] leads to decrease in tensile yield and ultimate strengths. For higher temperatures in Fe–17.5/20Cr-17.5/20Mn–0.59N and in Fe–18Cr–21.5Mn–1.5Ni–0.6/0.9N respectively, Moon et al. [21] and Xu et al. [22] observed the same variation. Holm et al. [23] also pointed out a decrease in room-temperature yield strength and ultimate tensile strength for an initially 33% cold-worked Fe–18Cr–19Mn–0.58N–0.06C submitted to annealing. After annealing for 24h between 500 °C to 1050 °C, it was observed that the strengths decrease with the increase in temperature. For a Fe–18Cr–18Mn–0.63N–0.06C steel, cold-worked up to 50%, and then aged at 850 °C, a decrease in room temperature tensile yield and ultimate strengths when ageing time increased is also recorded by Feng et al. [24]. On Fe–18Cr–19Mn–0.9N–0.06C, Vanderschaeve et al. [25] also extensively studied the effect of annealing on a non-cold worked sample for temperatures between 400 °C to 900 °C. Compared to the initial hardness of 255 Hv, they only observed a small increase in hardness at 800 °C. This hardness also slightly increases with the annealing time, up to 305 Hv. After annealing at 800 °C, they observed the creation of the sigma phase. However, they attributed the hardness increase mainly to the discontinuous precipitation of the Cr_2_N phase.

### 1.3. Cr_2_N Precipitation

The creation of Cr_2_N precipitates is a well-known phenomenon that occurs in such high-N austenitic alloys. To avoid them, as specified by Rasouli et al. [26] for Fe–17Cr–11Mn–0.2N–0.2C, a thermal treatment at about 1000–1100 °C, followed by a rapid quenching is usually done, taking into account that the kinetics of the Cr_2_N precipitation is low enough to prevent it at these temperatures. This allows getting a pure austenitic alloy without precipitates. After ageing at temperatures near 800 °C, and for the high-N Fe–18Cr–19Mn–0.9N–0.06C alloy, Vanderschaeve et al. [25] extensively analyzed the discontinuous (also called cellular, or nitrogenous pearlite) precipitation of Cr_2_N. They observed that in an undeformed sample, this precipitation occurs on austenitic γ grain boundaries using a quasi-pearlitic reaction γ → γ’+ Cr_2_N. Cr_2_N precipitates form cells showing alternative lamellae of Cr_2_N and a new γ’ austenitic phase. The Cr_2_N phase has some crystallographic orientation relationships with the initial austenite. In their alloy, the chemical composition of the precipitates is (Cr_0.73_Fe_0.17_Mn_0.10_)_2_N. The new γ’ austenite has a lattice parameter equal to 0.3608 nm, lower than that of initial austenite. Vanderschaeve explained this discontinuous precipitation by the diffusion of nitrogen, which is easy because nitrogen is an interstitial atom, so the diffusion of N is a long-range volume diffusion mechanism. As Cr is a substitutional element, its diffusion is much slower and should occurs along grain boundaries. Moon et al. [21] observed in a Fe–17.5/20Cr–17.5/20Mn–0.59N alloy that, during hot working at 800 °C, some small intergranular Cr_2_N particles are created, promoting cracks. Feng et al. [24] studied the effect of cold deformation prior to ageing at 850 °C on Cr_2_N precipitation in a Fe–18Cr–18Mn–0.63N alloy. They observed for high deformation that no cellular precipitation exists even for an 8 h or longer ageing, but Cr_2_N precipitates as granular and massive particles inside grains.

### 1.4. Aim of the Paper

This paper intends to analyze the effects of annealing at a moderate temperature, together with the effect of the initial cold working ratio of a Biodur 108© material. The annealing temperature is 575 °C. Samples with various annealing times and various initial cold working ratios are studied. In order to evidence the synergistic effect of these two parameters, the results are divided into five parts.

The first part is devoted to the analysis of the as-received Biodur 108© material. The second part evidences the effect of cold-working and annealingon hardness. In the following parts, microstructural, chemical and crystallographic analyses are performed, in order to explain the evolution of hardness. Therefore, in the third part, the effect of annealing alone is presented, using a weakly deformed sample. Then, the fourth part is devoted to the annealing of highly cold-worked materials. In such materials, and for long periods of annealing, the microstructure is fully changed, corresponding to high hardness levels. The last part is dedicated to the understanding of the mechanisms responsible for the microstructural changes. For that purpose, the behavior of intermediate cold working level samples depending on the duration of annealing is then investigated.

## 2. Materials and Methods

### 2.1. Material and Treatment

The material studied in this work is cold-deformed (25%) commercial Biodur 108© (stainless steel—UNS S29108) in wire shape with Ø 3.17 mm and provided by L. Klein SA. The certified composition is given in Table 1 and was verified by scanning electron microscopy with energy dispersive X-ray spectroscopy (SEM-EDX) analysis.

The samples machined from the as-received material were further cold-rolled to two total reduction ratios of 42% and 85%. The cold-rolled samples were then annealed at 575 °C in an air-furnace for various times: from 0 h to 978 h (40.75 days).

The reduction ratio *R* is calculated from the usual Formula (1):

(1) $$R=\frac{S_{0}-S}{S_{0}}$$

where *S*_0_ is the initial section and *S* the section after deformation.

The commercial alloy has been cold drawn (25%) by the company in order to obtain Ø 3.17 mm wires. In this study, the wire has been cold rolled in order to reach total calculated reduction ratios of 42% or 85%.

**Figure 1:**
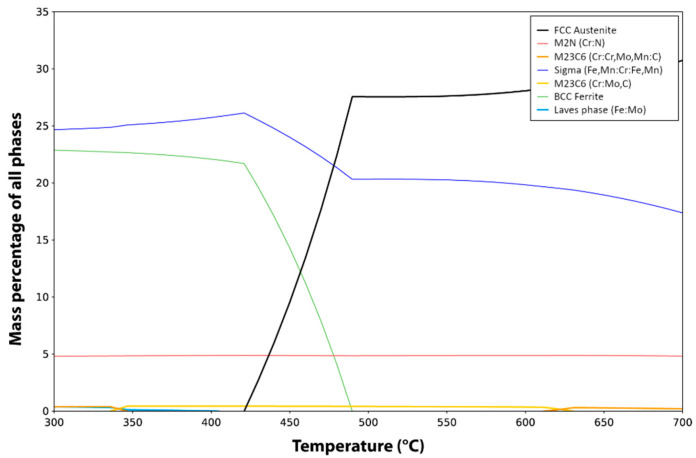
Equilibrium phases at various temperatures according to Thermo-Calc calculations. Black line is FCC austenite γ’, red line is a trigonal Cr_2_N type phase and blue line is a body-centered tetragonal (BCT) σ phase.

Calculus with the Thermo-Calc software (v. 2021b) and the TCFE9 database allows knowing the equilibrium phases expected after a 575 °C long time heat treatment. The equilibrium phases in the 300–700 °C temperature range are presented in Figure 1, and Table 2 gives the composition of the main expected phases at 575 °C. From Figure 1 and Table 2, one can deduce that the main equilibrium phases are austenite γ’, with almost no concentration of N, a σ phase, and a Cr_2_N type phase.

### 2.2. Investigation Methods

The crystal structures of the phases in the treated samples were identified by X-ray diffraction measurements. X-ray diffraction spectra were recorded in the 40–90° 2θ range by diffraction on polished samples using a Co-Kα anode (λ = 0.1789 nm) on a 4 circle Bruker D8 diffractometer using a plane detector.

The microstructural and crystallographic orientation features of the treated samples were examined by scanning electron microscopy (SEM)—including imaging, EDX, electron backscattering diffraction (EBSD) and transmission Kikuchi diffraction (TKD) measurements -, as well as by transmission electron microscopy (TEM). The samples were first mechanically polished, then polished with a ¼ µm diamond paste and finished by an oxide polishing suspension (OPS). The microstructural features were examined using a Zeiss Supra 40 SEM in the backscattered election (BSE) imaging mode. The chemical composition was analyzed in the same SEM equipped with a Bruker X Flash 6/30 detector. The composition was quantitatively measured at 15kV using the standards of pure Fe, Mn, Mo, Cr and BN, the latter being a reference for nitrogen (registered MAC standards n° 7478). The crystallographic orientation features were examined by EBSD measurements at 15 kV with a JEOL F100 SEM equipped with an EBSD camera (Symmetry) and the Aztec software package (Oxford Instruments).

A TEM foil sample was prepared by Ga ion milling (FIB) using a Zeiss Auriga 40 FIB- SEM. The crystallographic orientation features were also examined on this foil by “on-axis” TKD measurements [27] in the Zeiss Supra 40 SEM using a Bruker Optimus detector at 30 kV coupled with EDX in order to acquire the chemical and crystallographic structure at the same time. The TKD measurements were performed with a step size of 20 nm and the EBSD measurements were conducted with a step size of 30 nm or 50 nm for high magnification scale and 300 nm for lower magnification. The substructure features and crystal structures of the phases of one heat treated sample were examined in a Philips CM200 TEM equipped with an LaB6 cathode, a Gatan Orius 833 charge coupled device (CCD) camera and homemade automatic orientation analysis software—Euclid’s Phantasies (EP) [28].

Vicker’s hardness was measured under a load of 1 kgf (9.81 N) on a Buehler Micromet 5104. The size of the hardness indentations stands between 45 to 80 µm. Each hardness value is averaged from six measurements.

## 3. Results

### 3.1. As-Received Material

**Figure 2:**
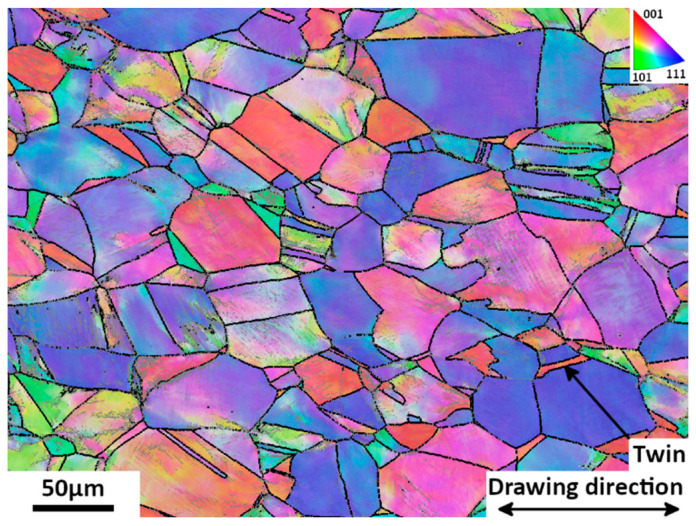
EBSD drawing direction Inverse Pole Figure (IPF) map of austenite with high angle boundaries (10°) outlined in black of the as-received 25% cold-drawn sample. Step size is 300 nm.

The microstructure of the as-received material is presented in Figure 2, corresponding to a 25% cold-drawn state. Clearly, the initial material consists of single austenite (γ phase) with an FCC structure and having a mean grain size of 33 µm. Some twins appear in the austenite grains which can be clearly seen in Figure 2. From EDX analyses, no chemical segregation was detected. The lattice parameter *a* of the austenite (γ phase) determined from the diffraction peaks in the X-ray spectrum is equal to 0.3633 ± 0.0001 nm. The initial hardness of this sample is 425 Hv. As already stated in previous studies [10], although yield strength has a high value, this material stays ductile.

### 3.2. Evolution of Hardness with Deformation and Annealing Time 

**Figure 3:**
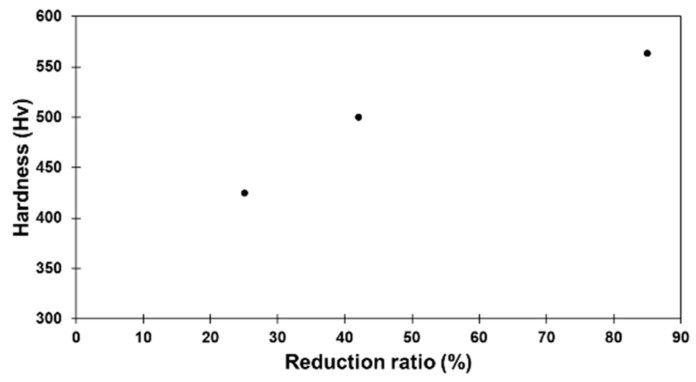
Evolution of mean hardness with rolling reduction before annealing. Error bar is smaller or about marker size.

**Figure 4:**
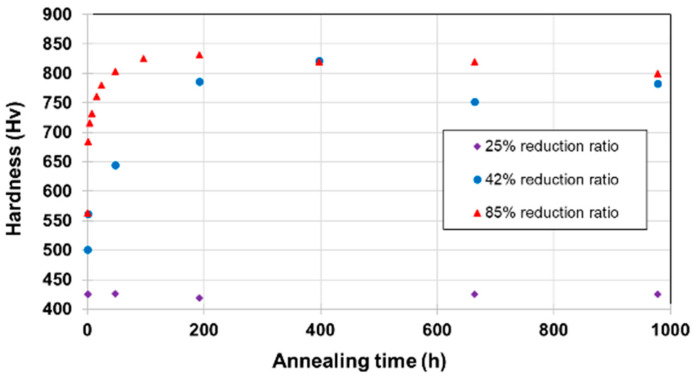
Evolution of mean microhardness with annealing times when annealed at T = 575 °C. Error bar is smaller or about marker size.

Figure 3 presents the measured hardness of the samples cold-rolled to different total reductions before annealing. It is observed that the hardness increases with the increase in deformation, reaching 550 Hv at the 85% reduction. This result is in line with what is reported in the literature [4,10]. Interestingly, after annealing, the hardness of the samples demonstrates two different tendencies depending on the intensity of the prior deformation, as shown in Figure 4. For the 25%-drawn (as-received) sample, annealing has no effect on hardness no matter how long the duration of the annealing. However, for the further deformed samples (42% and 85%), secondary hardening occurred. Hardness drastically increased when annealing started and then stabilized after a certain time. The increases are of about 64% for the 42%-rolled samples and of about 47% for the 85%-rolled ones with a maximum hardness as high as 830 Hv for the 85%-rolled sample, after 192 h of annealing. 

From these results, one can see that, although the annealing has a very low hardening effect on the as-received (lowly deformed 25%-drawn) material and some moderate effect on the further cold-rolled state (42%-rolled: Hv 504 and 85%-rolled: Hv 564) before annealing, a strong synergetic hardening appeared when combining deformation and annealing. The hardening occurs very quickly upon annealing with a pronounced increase during the first hours. After this stage, the hardness stabilizes with a slight decrease after 200 h of annealing. 

**Figure 5:**
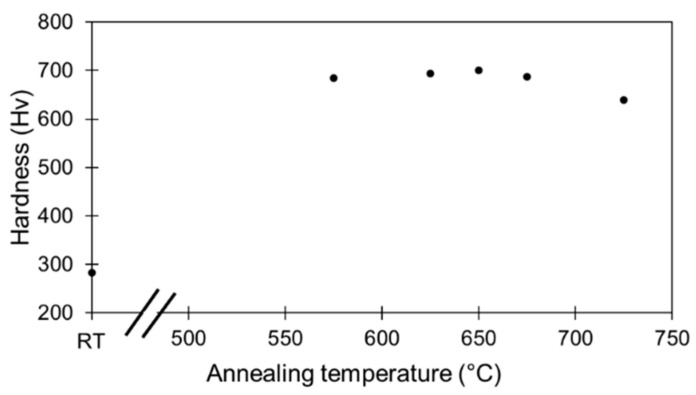
Evolution of mean microhardness of 85%-rolled sample with annealing temperatures for 1h annealing. Error bar is smaller or about marker size.

This synergetic hardening was observed for a limited temperature range, up to 650 °C. When annealing temperature was increased above 650 °C, the hardness gain decreased, as indicated in Figure 5. In order to study the mechanisms involved in such a hardness gain, the focus was only put on one annealing temperature, namely 575 °C.

### 3.3. Microstructure: Evolution of As-Received Sample with Annealing Time

**Figure 6:**
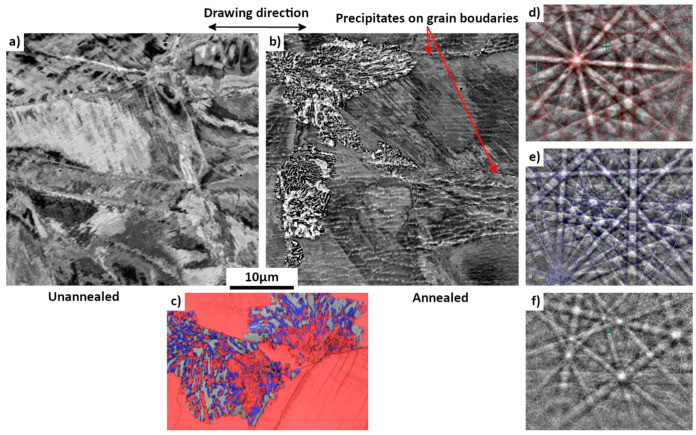
SEM BSE micrographs of differently treated samples. (**a**) as-received sample (25%-drawn). (**b**,**c**) 25%-rolled sample after 978 h annealing at 575 °C. (**c**) SEM EBSD micrograph. In (**c**) red: austenite phase, blue: Cr_2_N and gray (band quality indexed contrast): unknown phase. Corresponding EBSD Kikuchi patterns of the three phases: (**d**) austenite, (**e**) Cr_2_N, (**f**) unknown phase. For austenite and Cr_2_N, calculated line patterns are superimposed on experimentally acquired patterns showing the excellent fit of the two.

**Figure 7:**
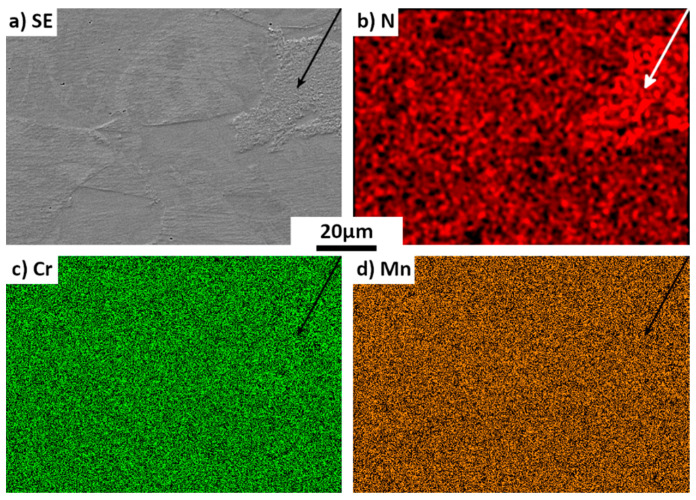
Micrograph and chemical maps of an area of as received sample (25%-drawn), annealed at 575 °C for 978 h. (**a**) secondary electron (SE) micrograph, (**b**–**d**) maps of the chemical element distribution of the same area. The arrows indicate the newly formed structure.

To better understand the effect of annealing on the differently treated Biodur 108© samples, the microstructure evolution was studied. Figure 6 shows the microstructure of the as-received (25%-drawn) sample after annealing at 575 °C for 978 h. For reference, the as-received state of the material is also presented in Figure 6a; its microstructure is composed of single austenite (γ phase). After the 978 h-annealing, a new structure appears in some areas, as seen in Figure 6b. Further EBSD analyses showed that the new structure is in cellular form with a mixture of three phases in lamellar shape, as shown in Figure 6c. The phase colored in red is the austenite phase with an FCC structure, the one in blue is Cr_2_N with a trigonal structure (space group: P$\bar{3}$1m) and the one in grey is a new unknown phase (detailed later). For reference, the EBSD Kikuchi patterns are also displayed in the figure (Figure 6d–f). For the two known phases, excellent fit can be seen between the experimental patterns and the calculated ones, confirming the crystal structures of the two phases. However, for the unknown phase, the Kikuchi lines are somewhat diffusive and cannot be indexed using the Inorganic Crystal Structure Database (ICSD). The morphology of this new structure is very similar to the cellular Cr_2_N/austenite mixture already described in high-N steels by Vanderschaeve et al. [25]. In the same time, austenite grain boundaries seem to be decorated by some very small precipitates (Figure 6b). Chemical maps presented in Figure 7 reveal that the new structure is associated with an enrichment in N. The N enrichment in the new structure is in fact associated with the formation of the Cr_2_N phase. 

Although the annealing temperature was quite low, the long annealing time allows some atom diffusion. As evidenced by the chemical maps in Figure 7, N-segregation appears after the long annealing, leading to the creation of N-rich areas, as indicated by the arrow in Figure 7b. The locations of the N-segregation coincide with the newly formed structure, as presented in Figure 7a. Although a long-range diffusion of N is clearly visible, no long-range diffusion of Cr happened, nor of Mn, as displayed in Figure 7c,d. This result is in agreement with the remarks already made by Vanderschaeve et al. [25].

Cr_2_N phase and unknown phase peaks were not observed on X-ray diffraction spectrum, which means that the volume fraction of these phases was low. The mean lattice parameter of the austenite does not change, compared to the non-annealed as-received material.

### 3.4. Microstructure of Highly Deformed Sample after Annealing

It is now interesting to study the 398 h and 978 h annealed microstructures of the 85%-rolled sample. With an 85% reduction ratio, hardness evolves with annealing time very quickly towards a maximum, as already presented in Figure 4, and then a slight decrease may be explained by coalescence mechanisms. Therefore, the hardness of the 978 h annealed sample is just slightly below the maximum.

**Figure 8:**
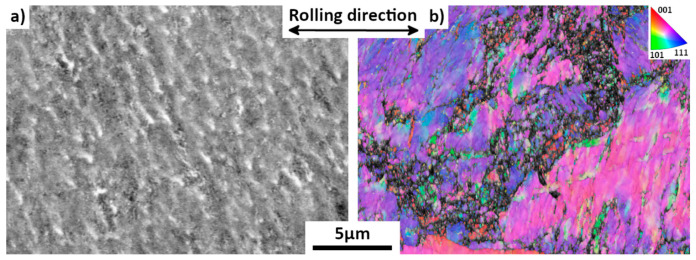
Un-annealed 85%-rolled sample. (**a**) BSE map. (**b**) EBSD IPF (//rolling direction) map (step size 30 nm).

**Figure 9:**
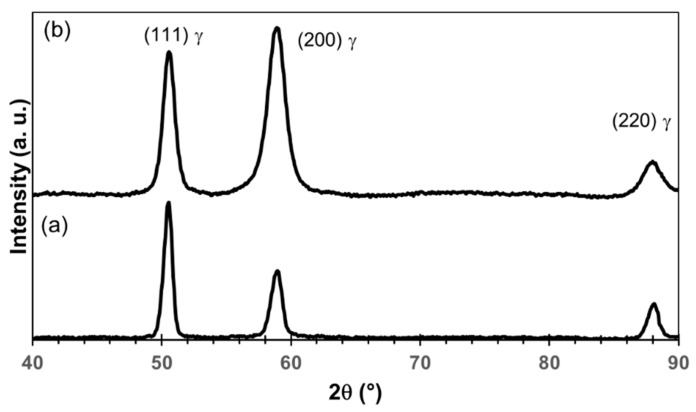
X-ray diffraction patterns of un-annealed (**a**) lowly-deformed and (**b**) 85%-rolled samples.

Figure 8 presents the 85%-rolled microstructure before annealing. Compared to the as-received sample (25%-drawn) presented in Figure 2, no clear grain boundaries are visible, and some wave-shaped substructure is present. From the X-ray diffraction spectrum (Figure 9b) and the EBSD maps, it is found that the sample is still composed of single austenite phase. However, this phase is heavily deformed, as evidenced by the broadening of the X-ray peaks with respect to those of the lowly-deformed as-received material, indirectly indicating that large numbers of crystal defects were created by the deformation. A large amount of mosaicity can be seen in Figure 8b inside austenitic grains. The mean lattice parameter of the austenite remains equal to 0.363 ± 0.001 nm. 

**Figure 10:**
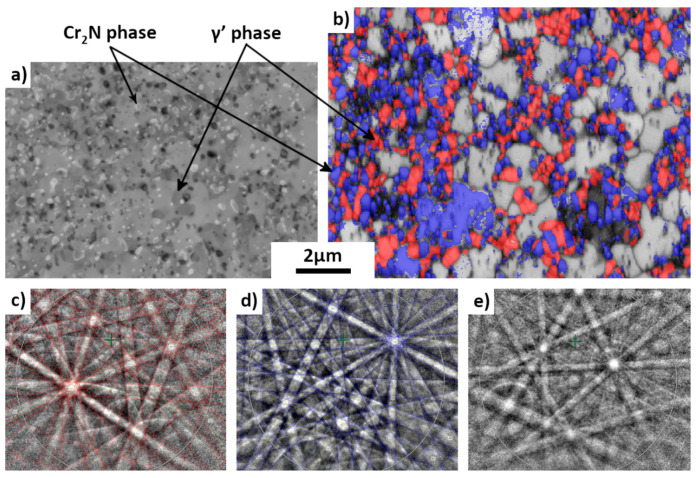
BSE (**a**) and EBSD (**b**) micrographs of same area of an 85% deformed sample after annealing at 575 °C for 398h. In EBSD micrograph (step size 50 nm), red: austenite phase, blue: Cr_2_N phase and gray (band quality indexed contrast): unknown phase. Corresponding EBSD Kikuchi patterns of the three phases: (**c**) austenite, (**d**) Cr_2_N, (**e**) unknown phase. For austenite and Cr_2_N, calculated line patterns are superimposed with experimentally acquired patterns showing the excellent fit.

When annealed at 575 °C for 398 h, the comparison between Figure 8 and Figure 10a revealed the fact that the microstructure changed drastically. The morphology of this new microstructure is very different from that of the 25%-drawn sample after long-time annealing. The former showed large austenite grains with some cellular shaped areas (Figure 6b and Figure 7a) with some lamellar mixture (Figure 6c), whereas the latter contains a mixture of different constituents with equiaxed morphologies and much smaller sizes as presented in Figure 10. The EBSD map confirms the existence of three phase constituents. As for nitrogenous cellular structure in the lowly deformed sample after long-time annealing (Figure 6c), it is seen that the new structure is still composed of three phases: Cr_2_N, austenitic phase and the unknown phase (the primary structure identification will be given later), as evidenced by their EBSD Kikuchi patterns in the figure. The austenitic phase observed in the highly deformed samples after long-time annealing is the so-called γ’ phase.

**Figure 11:**
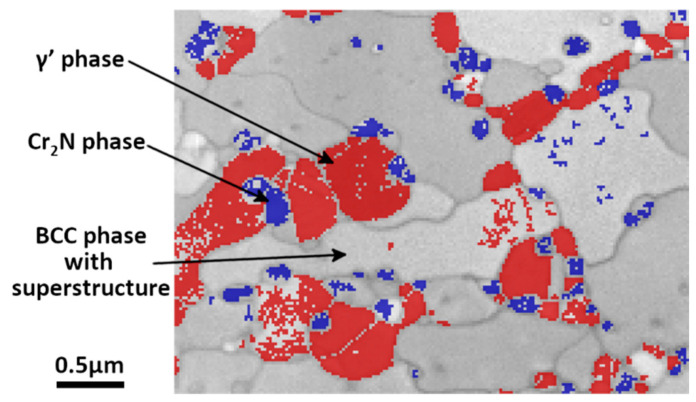
TKD phase index micrograph of an 85%-rolled sample after annealing at 575 °C for 978 h. Blue: Cr_2_N; red: austenite γ’; gray (band quality indexed contrast): unknown phase.

**Figure 12:**
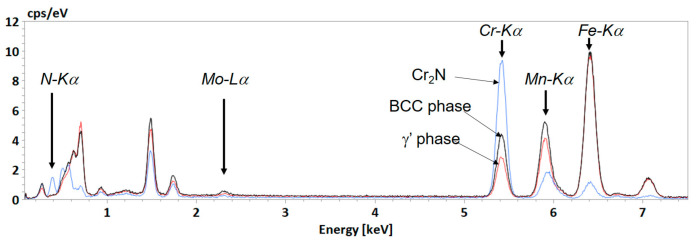
Chemical EDX spectra acquired on some characteristic points on the thin foil. Blue spectrum is recorded in chromium nitride phase, black spectrum in unknown phase, and red one in γ’ austenite.

As the precipitates are very small, the microstructure of the 85%-rolled sample after 978 h-annealing at 575 °C was further analyzed by TKD. The TKD micrograph is shown in Figure 11. It is seen that most of the grains of the unknown phase are of micrometric sizes, whereas the Cr_2_N is in particle shape with much smaller sizes (about 200 nm) and the γ’ phase is in smaller quantity with also smaller sizes in sub micrometer range. The chemical composition of the three phases was analyzed by SEM EDX in the same thin foil (Figure 12). Although a quantitative analysis cannot be reached, the results are representative of the phase composition because of the reduction of the interaction volume. It is revealed that the blue phase is rich in Cr and N but poor in Mn and Fe, confirming the result of the EBSD measurement. This phase is indeed the Cr_2_N phase.

**Figure 13:**
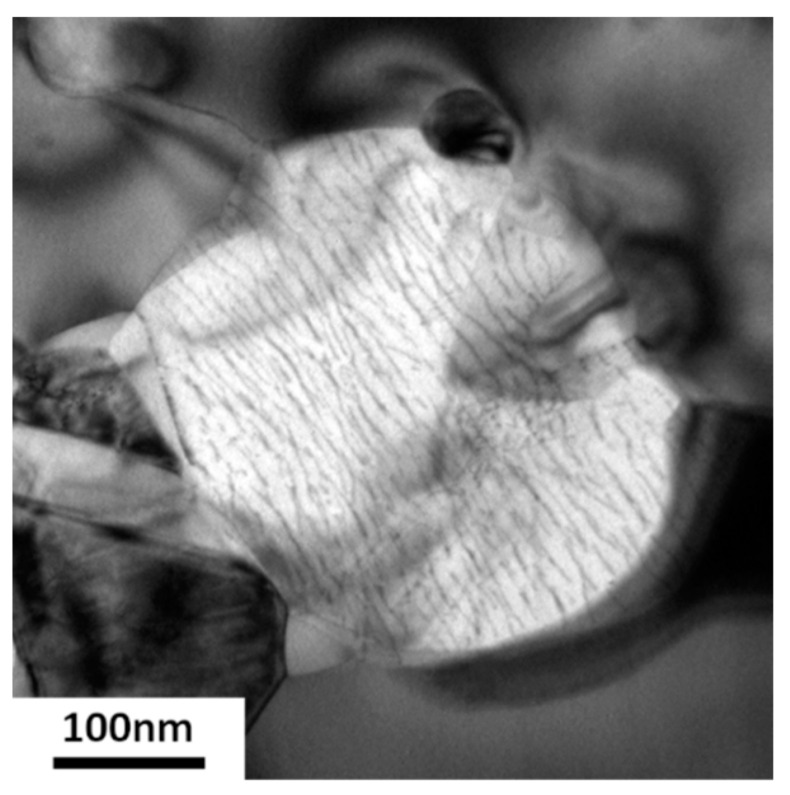
TEM bright field micrograph showing the dislocation arrays in γ’ grains in 85%-rolled sample after annealing at 575 °C for 978 h.

**Figure 14:**
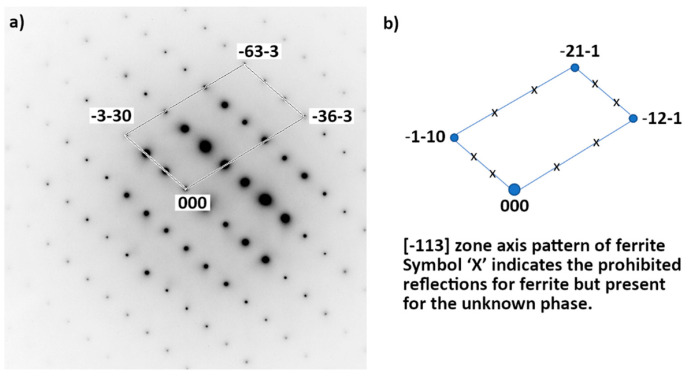
(**a**) TEM Selected Area Electron Diffraction (SAED) of the unknown phase in 85%-rolled sample after annealing at 575 °C for 978 h. It has a BCC superstructure. Incident beam is parallel to [-113]. (**b**) For reference, [-113] zone axis pattern of ferrite.

Combining the data given by all the techniques (EDX, EBSD, TEM, X-ray diffraction), the three phases can be well described:

(i) Chromium nitride has a trigonal symmetry P$\bar{3}$1 m. According to chemistry and crystallography, these precipitates are identified as (Cr_x_Mn_y_)_2_N, with a much higher quantity Cr, compared to the quantity of Mn. From the X-ray diffraction analysis, it is found that the lattice parameters are *a* = *b* = 0.477 nm and *c* = 0.4466 nm, instead of the theoretical parameters *a* = *b* = 0.48 nm and *c* = 0.4472 nm for the stoichiometric Cr_2_N. Although they are too small to be correctly chemically analyzed, one can estimate from the equilibrium calculi (Table 2) that these nitrides could be (Cr_1.66_Mn_0.34_Mo_0.06_)N.
(ii) The observed γ’ austenite has an FCC structure with a lattice parameter equal to 0.3592 ± 0.0003 nm deduced from its X-ray diffraction spectrum. While the deformed initial austenite in un-annealed samples contains about 1 wt% nitrogen and has a lattice parameter equal to 0.363 nm, no (or very little) N was found in this austenite. The correlation between lattice parameter and the concentration of N is well documented for austenite (Figure 13 in [25]) and confirms that no (or very little) nitrogen remains in the γ’ austenite. Although the chemical analysis may be only semi-quantitative due to the smallness of the grains, this phase is assumed to contain between 13 wt% and 19 wt% Cr, about 19–21 wt% Mn and a balance of Fe. Further TEM examination showed that, in the γ’ phase, there are large numbers of dislocation arrays, as indicated in Figure 13.
  Equilibrium calculi confirm that almost no N is expected in this phase. The observed concentration of Mn is also in agreement with the equilibrium data. This concentration of Mn is not far from the initial austenite Mn concentration (22.8 wt%). However, a strong discrepancy is observed for Cr concentration: 7.2 wt% are expected according to the equilibrium calculi, whereas more than 13 wt% Cr were observed (concentration of Cr in the initial austenite is 21.8 wt%).
(iii) The unknown phase is found to have a very complex crystal structure whose determination will be the focus of a separate study. It contains about 20–21 wt% Cr, 25 wt% Mn (slightly increased from the initial γ phase) and the balance of Fe. Almost no N was detected. Its crystal structure was further analyzed by TEM Selected Area Electron (SAED) diffraction, as shown in Figure 14. Diffraction patterns along more than five zone axes have been recorded and confirmed that the unknown phase possesses a BCC superstructure with the lattice parameter *a* of about 0.9 nm.

It should be noted that this phase is not the reported σ phase in the literature and in the equilibrium calculi, although the lattice parameter is close to *a_σ_* and *b_σ_* parameters (*a_σ_* = *b_σ_* = 0.8800 nm and *c_σ_* = 0.4544 nm), and like the *σ* phase, the unknown phase is also enriched in Mn (*σ* phase: 27.9 wt%, unknown phase: 25 wt% and nominal concentration of the material: 22.8 wt%). However, the crystal structure is different (*σ* phase: body-centered tetragonal, unknown phase: body-centered cubic) and the Cr concentration also differs (*σ* phase: 28.4 wt%; unknown phase: 20–21 wt%; nominal concentration: 21.8 wt%). The volume fraction is also different, as this unknown phase represents the major part of the material, instead of only 36.7% for *σ* phase in the equilibrium state.

Table 3 synthetizes the chemical measurements for the main chemical elements in the different phases.

As confirmed by EBSD measurements, X-ray diffraction and BSE maps, when the sample is 85% cold-rolled, these phases appear very early (observed at 1 h) during the heat treatment, concomitantly with the increase in hardness (c.f. Figure 4).

### 3.5. Microstructure: Evolution of Mediumly-Rolled Sample after Annealing

For the above study, the 85%-rolled sample was too severely deformed, as shown in Figure 8, thus it is difficult to reveal the mechanism of the formation of the new annealed structure. For the as-received sample (25%-drawn), the formation of the new structure did not occur intensively. A moderately-deformed state may be helpful. Therefore, the formation of the annealed structures of the 42%-rolled samples was then examined in order to understand the mechanism leading to the creation of the new structures.

**Figure 15:**
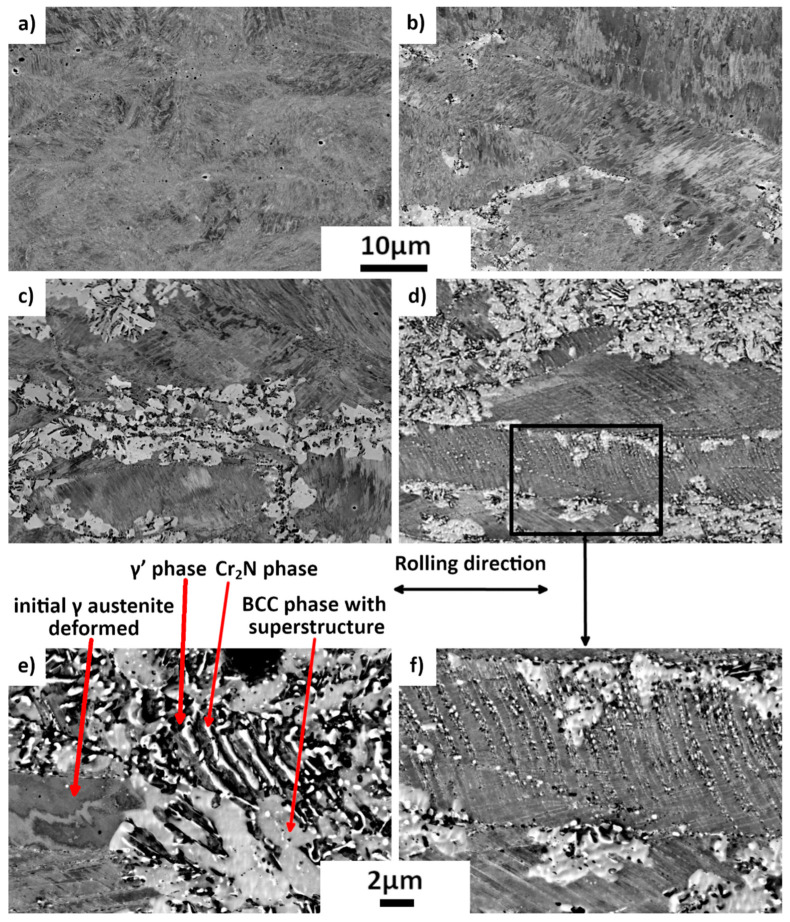
Evolution of microstructure with annealing times of 42%-rolled samples. SEM BSE micrographs. (**a**) 1 h; (**b**) 48 h; (**c**) 398 h; (**d**) 978 h; (**e**) and (**f**) magnified micrographs of 978 h-annealed samples. Cr_2_N precipitates may appear in white.

Figure 15 presents the evolution of the microstructures of the 42%-rolled samples after annealing at 575 °C for periods varying from 1 h to 978 h. It is seen in Figure 15a that after 1 h of annealing the microstructure is still dominated by the deformed γ grains elongated along the rolling direction and contains deformation bands. After the 48 h annealing (Figure 15b), the new annealed structures (white and black areas) appear preferentially along the γ grain boundaries. Thus, the new structure should contain the three different phases: γ’, Cr_2_N and the unknown phase. Cr_2_N precipitates have a globular shape. After further annealing (398h, Figure 15c and 978 h, Figure 15d), the proportion of the new structures increases and these structures gradually invades the whole sample. After the 978 h annealing, new phases are even generated on deformation bands (almost vertical in Figure 15d,f), still in globular shape. 

X-ray diffraction spectra confirm that the same phases appear during annealing in the 85%- and in the 42%-rolled samples. That is to say, white areas observed on Figure 15 are anticipated to be the new austenite γ’ and the unknown phase, and black precipitates are the Cr_2_N phase. 

After the 978 h annealing, very few cellular structures similar to the nitrogenous pearlite and already observed in the 978 h-annealed 25%-drawn sample can even be found (Figure 15e). As these structures were not found in short-time annealed sample, this lead to assume that the cellular structure corresponds to the return to the equilibrium state.

**Figure 16:**
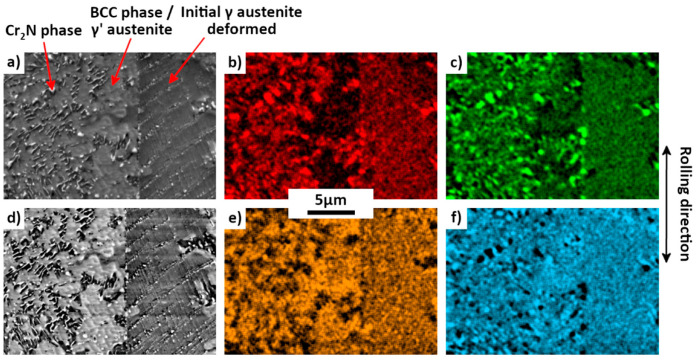
SEM-SE (**a**) and BSE (**d**) micrographs and SEM EDX micrographs (**b**,**c**,**e**,**f**) of a 42%-rolled sample after annealing at 575 °C for 978 h.

The chemical analysis by SEM EDX, presented in Figure 16, of the 42%-rolled sample after 575 °C annealing for 978 h confirms that, except for the Cr_2_N precipitates, the new phases (γ’ and BCC phase) are nearly free of N, in contrast to the initial austenite γ where the concentration of N is of about 1 wt%. An increase in the Mn concentration is also evidenced for the unknown phase. A sharp decrease in Mn and Fe inside the Cr_2_N precipitates is also detected.

## 4. Discussion

The present study was carried out to explain the increase in hardness observed when annealing is applied to a cold-worked high-N stainless steel. This increase is opposite to what was previously observed on austenitic high N stainless steels, as reported before [23,24]. Using complementary techniques, such as chemical analyses, EBSD mapping and MET examination, allows explaining how and why hardness increases with annealing. Such hardness increase was not observed for un-deformed annealed sample. Conversely, cold working alone does not allow reaching such hardness values. 

Many mechanisms are assumed to be involved. The formation of Cr_2_N precipitates during annealing is evidently associated with the increase in hardness. As they are small and homogeneously distributed due to the initial cold working, these precipitates act in an efficient way to slow down dislocations motion. Other mechanisms should also be involved. On one hand, γ austenite retains arrays of dislocations which may contribute to hardening. On the other hand, the new phase with a BCC super-structure should also impose difficulty to dislocation slip. For that reason, such phases are evidently assumed to be hard. The major contribution to hardening is also the submicronic or micronic size of the grains of the new phases, according to the Hall–Petch law.

During the annealing of the cold-rolled samples, these new phases are created along the grain or sub-grain boundaries. They may also be created onto deformation bands. This presence of crystalline defects, such as grain boundaries and dislocations, accelerates the formation of chromium nitride and other new phases and prevents cellular (nitrogenous pearlite) precipitation that occurs during the long-time annealing of lowly or moderately-deformed samples. Even for a short time, the same mechanism is already present.

One can notice that the 42% cold-rolled samples as well as the 85% cold-rolled samples reach similar hardness during annealing. This is quite surprising because the proportion of new BCC phase is smaller in 42% cold-rolled samples. However, for both types of samples, Cr_2_N precipitates are evenly distributed, even in the initial deformed austenite, as they are being created onto deformation bands. It may be concluded that they bring an essential contribution to the sample hardness increase.

Clearly, cold rolling, by creating lattice defects, promotes both the chemical diffusion and the nucleation of the new phases during further annealing. The cold-rolling increases phase-transformation kinetics. The chemical diffusion is increased due to the preferential path of diffusion created by the defects, and the nucleation of the new phases is increased due to a lower surface energy in the defect areas.

From the chemical analyses, it is concluded that the chemical diffusion plays a major role in the creation of the various phases. At 575 °C, nitrogen, as an interstitial atom, can diffuse in volume to long distances provided that the annealing time is sufficient, in such a way that all the material is depleted in nitrogen except in the precipitates. The equilibrium structure is then composed of nitrogenous perlite with lamellar Cr_2_N. For the cold-rolled material, the diffusion of nitrogen may be enhanced along grain or sub-grain boundaries as well as along dislocations, leading to short-range diffusion and allowing for the creation of globular Cr_2_N precipitates at shorter annealing times. 

However, at 575 °C, Cr and Mn diffusions are very slow and should dictate the formation of the new phases. For a lowly deformed sample, no long-range diffusion of these elements was found, even for a long time annealing. Small fractions of nitrogenous perlite are created with only short-range diffusion of Cr and Mn, giving birth to cellular structures with very thin lamellae. These cellular structures are widely spaced and do not contribute to secondary hardening.

In cold-rolled material, the existence of sub-grain boundaries and dislocations is assumed to accelerate Cr and Mn diffusion. This allows for the formation of the globular Cr_2_N precipitates. Locally, γ’ austenite slowly grows poor in Cr in order to create the Cr_2_N precipitates. Mn is pushed away from Cr_2_N towards the unknown BCC phase which is then slightly enriched in Mn. The γ’ austenite does not reach the equilibrium composition, as it is not depleted enough in chromium. Similarly, the unknown BCC phase does not reach the equilibrium composition either, as it is not enriched enough in manganese and in chromium compared to the σ equilibrium phase. 

This change in compositions may be the reason why this BCC phase is created instead of the BCT σ phase. The unknown phase may be a metastable one. Could the unknown phase come from a distortion of the σ phase, due to the incomplete enrichment in Mn and Cr? How to explain its superstructure? These questions are still open, and are now under investigation.

As this unknown phase already exists in the long-time annealed low-deformed sample (see Figure 6c), it seems that, at this temperature, the nitrogenous pearlite is formed from the reaction: γ → γ’ + Cr_2_N + unknown phase, instead of the reaction that is commonly accepted, that is to say γ → γ’+ Cr_2_N. 

During long-time annealing, the hardness evolves toward a plateau or slightly decreases. One reason could be that some coalescence mechanisms intervene, as the material tries to reach its equilibrium state. It is assumed that grain sizes increase during annealing, thus leading to decrease in hardness. In very long-time annealing, globular Cr_2_N precipitates tend to come back to the lamellar shape of the nitrogenous perlite, contributing to the slight hardness decrease.

## 5. Conclusions

(1) A secondary hardening mechanism was identified for the Biodur 108© material, when annealed at 575 °C, after cold deformation.
(2) When the material is cold-deformed and annealed, the creation of homogeneously distributed globular Cr_2_N is observed. For a long annealing time, these precipitates were also created onto deformation bands. They are assumed to be mainly responsible for the secondary hardening.
(3) A new superstructured BCC phase is discovered. Its crystalline structure is complex and will be the focus of a forthcoming paper. This phase is almost free of N and enriched in Mn.
(4) Together with the very small globular Cr_2_N precipitates and the small grains of the BCC unknown phase, very small grains of an austenite with a very low concentration of N are observed in the rolled and annealed samples.
(5) The nitrogenous perlite observed after long-time annealing at 575 °C of a low-rolled sample, is constituted of three types of lamellae: Cr_2_N, unknown BCC phase and austenite.
(6) The secondary hardening is then explained by the combination of several mechanisms:
  − the creation of small and very small Cr_2_N precipitates.
  − the submicronic (or few microns) size of the new phases.
  − the fact that the main part of the newly created microstructure is the superstructured phase.

## Figures and Tables

**Table 1:** Chemical composition, in wt%, of Biodur 108© material.

	C	Mn	Cr	N	Mo	Si	Fe
Certified Composition wt %	0.042	22.76	21.34	1.03	0.71	0.16	Bal.
Measured by SEM-EDX wt(%)		22.8 ± 0.4	21.8 ± 0.4	1.1 ± 0.7	0.72 ± 0.03	0.27	Bal.

**Table 2:** Composition (wt%), volume, and mass fraction of the main equilibrium phases at 575 °C, obtained from Thermo-Calc calculi with the TCFE9 database.

Phase	Mass Fraction	Volume Fraction	Mn	Cr	N	Mo	Si	Fe
austenite γ′	52.27%	50.85%	20.7	7.2	0.007	0.09	0.3	71.7
σ phase	37.84%	36.70%	27.9	28.4	0	0.3	0.009	43.4
Cr_2_N type	9.18%	11.74%	15.0	69.3	11.2	4.5	0	0

**Table 3:** Concentrations (wt%) of the main chemical elements in the different phases and comparison with equilibrium composition. In bold are the measured data having discrepancies with the equilibrium compositions. The value with (*) has been measured in a SEM with a wavelength dispersive X-ray analysis (WDX), using a LSM80E Ni–C crystal, at 5 kV in order to reduce the interaction volume.

Phase		Mn	Cr	N	Fe
austenite γ	*initial composition*	22.76	21.34	1.03	53.96
austenite γ’	*Measured (semi-quantitative)*	19–21	**13–19**	≈0	Bal.
	*Equilibrium calculi*	20.7	7.2	0.007	71.7
unknown BCC phase	*Measured (semi-quantitative)*	**25**	**20–21**	≤0.06 *	Bal.
σ phase	*Equilibrium calculi*	27.9	28.4	0	43.4
Cr_2_N type	*Equilibrium calculi*	15.0	69.3	11.2	0

## Data Availability

The raw/processed data required to reproduce these findings cannot be shared at this time as the data also form part of an ongoing study.

## References

[1] Uggowitzer P., Magdowski R., Speidel M.O. (1996). Nickel Free High Nitrogen Austenitic Steels. ISIJ Int..

[2] Li H.-B., Jiang Z.-H., Zhang Z.-R., Xu B.-Y., Liu F.-B. (2007). Mechanical Properties of Nickel Free High Nitrogen Austenitic Stainless Steels. J. Iron Steel Res. Int..

[3] Jung Y.-S., Kang S., Jeong K., Jung J.-G., Lee Y.-K. (2013). The effects of N on the microstructures and tensile properties of Fe–15Mn–0.6C–2Cr–xN twinning-induced plasticity steels. Acta Mater..

[4] Balachandran G., Bhatia M.L., Ballal N.B., Rao P.K. (2000). Influence of Thermal and Mechanical Processing on Room Temperature Mechanical Properties of Nickel Free High Nitrogen Austenitic Stainless Steels. ISIJ Int..

[5] Talha M., Behera C., Sinha O. (2015). Effect of nitrogen and cold working on structural and mechanical behavior of Ni-free nitrogen containing austenitic stainless steels for biomedical applications. Mater. Sci. Eng. C.

[6] Murata Y., Ohashi S., Uematsu Y. (1993). Recent Trends in the Production and Use of High Strength Stainless Steels. ISIJ Int..

[7] Behjati P., Kermanpur A., Najafizadeh A., Baghbadorani H.S., Karjalainen P., Jung J.-G., Lee Y.-K. (2014). Design of a new Ni-free austenitic stainless steel with unique ultrahigh strength-high ductility synergy. Mater. Des..

[8] Behjati P., Kermanpur A., Najafizadeh A., Baghbadorani H.S., Karjalainen P., Jung J.-G., Lee Y.-K. (2014). Effect of Nitrogen Content on Grain Refinement and Mechanical Properties of a Reversion-Treated Ni-Free 18Cr-12Mn Austenitic Stainless Steel. Met. Mater. Trans. A.

[9] Rasouli D., Kermanpur A., Ghassemali E., Najafizadeh A. (2019). On the Reversion and Recrystallization of Austenite in the Interstitially Alloyed Ni-Free Nano/Ultrafine Grained Austenitic Stainless Steels. Met. Mater. Int..

[10] Carpenter Technology, Technical Data Sheet. https://www.carpentertechnology.com/hubfs/7407324/Material%20Saftey%20Data%20Sheets/Biodur%20108.pdf.

[11] Patnaik L., Maity S.R., Kumar S. (2020). Status of nickel free stainless steel in biomedical field: A review of last 10 years and what else can be done. Mater. Today Proc..

[12] Gavriljuk V., Petrov Y., Shanina B. (2006). Effect of nitrogen on the electron structure and stacking fault energy in austenitic steels. Scr. Mater..

[13] Lee T.-H., Shin E., Oh C.-S., Ha H.-Y., Kim S.-J. (2010). Correlation between stacking fault energy and deformation microstructure in high-interstitial-alloyed austenitic steels. Acta Mater..

[14] Qi-Xun D., An-Dong C.X.-N.W., Xin-Min L. (2002). Stacking fault energy of cryogenic austenitic steels. Chin. Phys..

[15] Mosecker L., Saeed-Akbari A. (2013). Nitrogen in chromium–manganese stainless steels: A review on the evaluation of stacking fault energy by computational thermodynamics. Sci. Technol. Adv. Mater..

[16] Saller G., Spiradek-Hahn K., Scheu C., Clemens H. (2006). Microstructural evolution of Cr–Mn–N austenitic steels during cold work hardening. Mater. Sci. Eng. A.

[17] Molnár D., Lu S., Hertzman S., Engberg G., Vitos L. (2020). Study of the alternative mechanism behind the constant strain hardening rate in high-nitrogen steels. Mater. Charact..

[18] Singh B.B., Sivakumar K., Bhat T.B. (2009). Effect of cold rolling on mechanical properties and ballistic performance of nitrogen-alloyed austenitic steels. Int. J. Impact Eng..

[19] Mosecker L., Pierce D., Schwedt A., Beighmohamadi M., Mayer J., Bleck W., Wittig J. (2015). Temperature effect on deformation mechanisms and mechanical properties of a high manganese C+N alloyed austenitic stainless steel. Mater. Sci. Eng. A.

[20] Terazawa Y., Ando T., Tsuchiyama T., Takaki S. (2009). Relationship between Work Hardening Behaviour and Deformation Structure in Ni-free High Nitrogen Austenitic Stainless Steels. Steel Res. Int..

[21] Moon J., Lee T.-H., Shin J.-H., Lee J.-W. (2014). Hot working behavior of a nitrogen-alloyed Fe–18Mn–18Cr–N austenitic stainless steel. Mater. Sci. Eng. A.

[22] Xu L.-W., Li H.-B., Jiang Z.-H., Cai M.-H., Jiao W.-C., Feng H., Zhang S.-C., Lu P.-C. (2020). Hot Deformation Behavior of P550 Steels for Nonmagnetic Drilling Collars. Steel Res. Int..

[23] Holm H., Uggowitzer P., Speidel M. (1987). Influence of annealing temperature on the microstructure and mechanical properties of a high nitrogen containing austenitic stainless steel. Scr. Met..

[24] Feng S., XiaoWu L., Yang Q., ChunMing L. (2013). Effects of Cold Deformation and Aging Process on Precipitation Behavior and Mechanical Properties of Fe-18Cr-18Mn-0.63N High-Nitrogen Austenitic Stainless Steel. Steel Res. Int..

[25] Vanderschaeve F., Taillard R., Foct J. (1995). Discontinuous precipitation of Cr2N in a high nitrogen, chromium-manganese austenitic stainless steel. J. Mater. Sci..

[26] Rasouli D., Kermanpur A., Najafizadeh A. (2019). Developing high-strength, ductile Ni-free Fe–Cr–Mn–C–N stainless steels by interstitial-alloying and thermomechanical processing. J. Mater. Res. Technol..

[27] Brodu E., Bouzy E., Fundenberger J.-J., Guyon J., Guitton A., Zhang Y. (2017). On-axis TKD for orientation mapping of nanocrystalline materials in SEM. Mater. Charact..

[28] Fundenberger J.-J., Morawiec A., Bouzy E., Lecomte J.-S. (2003). Polycrystal orientation maps from TEM. Ultramicroscopy.

